# Genome-wide analyses of genomic diversity, population structure and selection signatures in Italian turkey populations

**DOI:** 10.1016/j.psj.2024.104543

**Published:** 2024-11-20

**Authors:** Medhat S. Saleh, Vincenzo Landi, Martijn F.L. Derks, Gerardo Centoducati, Martien A.M. Groenen, Pasquale De Palo, Elena Ciani, Maria G. Strillacci, Alessandro Bagnato, Nicola Pugliese, Elena Circella, Antonio Camarda

**Affiliations:** a Department of Veterinary Medicine, University of Bari Aldo Moro, 70010 Valenzano, Italy; b Animal Breeding and Genomics, Wageningen University & Research, P.O. Box 338, Wageningen, 6700 AH, the Netherlands; c Department of Animal Production, Faculty of Agriculture, Benha University, Benha 13736, Egypt; d Department of Biosciences, Biotechnologies and Environment, University of Bari Aldo Moro, Bari, Italy; e Department of Veterinary Medicine, Università degli Studi di Milano, Via dell'Università 6, 26900, Lodi, Italy

**Keywords:** Genome-wide scan, Genetic diversity, Population structure, Selection signature, Turkey

## Abstract

Italian local turkey populations are an important source of genetic diversity that should be preserved through an in vivo approach. Whole genome sequencing (WGS) and genotyping datasets were used to assess genetic variability within and across populations, to perform a genome-wide comparative analysis among populations and to identify selection signatures in Italian turkey populations. We used new data from 73 WGS samples (12X) representing five turkey populations, together with previous data from 107 birds genotyped with the Affymetrix 600K single nucleotide polymorphism (SNP) turkey array from 11 populations. The PCA and Admixture show a relatively strong isolation effect between the populations. Moreover, the values of genomic inbreeding based on ROH (*F*_ROH_) showed marked differences among populations and ranged from 0.096 to 0.643. Selective sweeps were identified using the integrated haplotype score (iHS) within the local group, the commercial line, and the Narragansett breed, resulting in the identification of 20, 19, and 27 regions with a total of 73, 48, and 90 candidate genes, respectively. Some of these genes such as *FAM107B, MSTN, PDZRN4, HSF2* and *GJA1* are associated with heat stress, growth, and carcass traits. We conclude that our results improve our understanding of the genomic architecture of the Italian turkey populations. The findings of iHS suggest that selection can play a significant role in shaping selection signatures in local turkey populations and could provide a basis for identifying gene mutations that may be beneficial in adaptation to climate change. Our results will be useful in developing and implementing conservation and selection plans for Italian turkey populations.

## Introduction

Domestication of wild turkey (*Meleagris gallopavo gallopavo*) originated in North America (Mock et al., 2002; Bernini et al., 2021) and was introduced into Europe by the Spanish conquistadors in the sixteenth century. The novelty and unique qualities of the meat subsequently allowed turkeys to spread rapidly throughout Europe. Notably, the turkeys’ introduction to Italy in 1520 and their rapid proliferation, underscore their integration into the country's agricultural and culinary heritage (De Grossi Mazzorin and Epifani, 2016; Maltin and Jakobsson, 2023). Bone remains suggest that turkeys were used for consumption as early as the1600s, being featured in culinary recipes of that time (Eiche, 2004; De Grossi Mazzorin and Epifani, 2016). Turkeys are the second main source of poultry meat and Europe has contributed 37.2% of the world's turkey meat during the past five years (FAO, 2023).

Italian-origin turkey populations are an important source of genetic diversity that should be preserved through an in vivo approach. Italian domestic turkey populations show wide phenotypic variation, including plumage color, body size and weight. Heritage populations can be considered as a source of genetic variability that should be maintained and exploited. These heritage populations have unique characteristics, in particular their ability to adapt to harsh environments and resistance to diseases (Colli et al., 2011; Marelli et al., 2022).

Recently in Basilicata and Puglia regions in south Italy some turkey populations (BAS-APU) raised in few farms in marginal areas have been discovered. There are different colors, buff, and black plumage with buff or white streaks and adult animals weigh up to 9 kg for males and 3.5 kg for females. The female produces 50/60 eggs per year. BAS-APU populations are rustic and adapted to harsh environments**.**

Single nucleotide polymorphism (SNP) of genotypic arrays and whole genome sequencing (WGS) datasets can be used to assess the comprehensive evolution of genomic variability within and across populations and make it possible to detect the genomic regions and genes that have been subjected to selection (Aslam et al., 2014; Strillacci et al., 2020; Bello et al., 2023).

The accessibility of high-density SNP arrays has also made it possible to examine the distribution of both homozygosity and heterozygosity within genomes of livestock species (Makanjuola et al., 2020; Bernini et al., 2021; Wang et al., 2022). Runs of homozygosity (ROH), which are segments of continuous homozygosity, help analyze animal genomes and comprehend the effects of powerful selection (Adams et al., 2021). ROH islands were utilized to assess genomic characterization and inbreeding coefficients estimation (Marras et al., 2018; Adams et al., 2021) or to identify selection signatures in turkey's genome (Marras et al., 2018; Bernini et al., 2021). Strillacci et al. (2020) reported that short ROH and ROH islands detected in commercial hybrid turkeys and Mexican breeds included genes associated with the percentage and weight of abdominal fat, eggshell color and yolk weight. Bernini et al. (2021) discovered a ROH island containing many genes that might have been under selection on chromosome 10 e.g. *PTGS2* and *PLA2G4A* genes associated with reproductive efficiency in all studied populations.

High-throughput sequencing and SNP genotyping technologies have significantly improved our ability to discover such selection signatures. Several methods for detecting selection signals have been developed, including site frequency spectrum (SFS)-based methods, linkage disequilibrium, and local variance minimization (Bello et al., 2023). Selective sweeps result in a high-frequency haplotype of variants at high linkage disequilibrium (LD) (Sabeti et al., 2002). LD-based techniques, such as integrated haplotype score (iHS), target long homozygous regions with high haplotype frequencies resulting from selection. SNP genotypic data has become the preferred method for detecting selection signals since it is less affected by ascertainment bias (Zhao et al., 2015; Mastrangelo et al., 2023).

Heritage turkey populations have been studied recently, providing some information about their genetic diversity (Aslam et al., 2012; Marras et al., 2018; Canales Vergara et al., 2019; Strillacci et al., 2021). Since turkey populations are raised on rural and family farms, the size of the turkey population is currently very limited. Therefore, more actions should be taken to preserve turkey's biodiversity. Investigation of genomic diversity, population structure and selection signatures of these populations may help in performing selection programs and conservation strategies. This study aims to investigate the genomic diversity and population structure, runs of homozygosity of Basilicata and Apulian turkey populations, to conduct a genome-wide comparative analysis among Italian local turkey populations, and to identify selection signatures across Italian turkey populations.

## Materials and methods

### Sampling, DNA extraction and sequencing

In 2023, a total of 73 blood samples (approximately 2 mL each) were collected from the wing veins of the Apulian C (APU-C; 5), Apulian M (APU_M; 7), Apulian PS (APU_PS; 8), Apulian PN (APU_PN; 7) and Basilicata (BAS; 46) populations. These samples belong to individuals originally collected from two regions (Puglia and Basilicata) in southern Italy, in five small farms. To our knowledge, these birds have not been subjected to any selection by their owners, who have allowed them to breed according to naturally occurring random mating as they are raised as a group in the backyard. There is no pedigree information available for these animals. A brief description of each turkey group including geographic region of the sample, feather color and adult body weight is provided in Table 1. The samples were stored in Vacutainers® tubes containing EDTA as an anticoagulant. These new samples were sent for DNA extraction and whole genome sequencing at Neogen (Ayr, Scotland, UK) following their internal procedures. The WGS data for all samples was generated using an Illumina platform with paired-end reads (read length ∼150 bp). The average sequencing coverage across samples is around 12x, with the majority of bases (>93%) having a quality score above Q30. Each sample was processed using the

**Table 1:** Population name, number of samples, body weight (kg) and plumage color, sampling region of the turkey populations included in the study.

Population	Code	N*		body weight		plumage color	Region
		Male	Female	Male	Female		
Apulian C	APU_C	1	4	3.5	7	Black with white streaks	Puglia, Italy
Apulian M	APU_M	2	5	3.5	7	Black with white streaks	Puglia, Italy
Apulian PN	APU_PN	2	5	4	8	Buff with white streaks	Puglia, Italy
Apulian PS	APU_PS	2	6	3.5	7	Black with white streaks	Puglia, Italy
Basilicata	BAS	10	36	4.5	9	Buff or black with white streaks	Basilicata, Italy
Total		17	56				

⁎ N, number of individuals per population.

DRAGEN pipeline, ensuring high-quality data for downstream analysis. We used 107 animal genotypes from the Axiom® Turkey Genotyping Array (Affymetrix, Santa Clara, CA, USA) containing 634,067 SNPs previously genotyped by Strillacci et al. (2019, 2020, 2021). These animals represent nine of the most common Italian local turkey populations (Brianzolo: 10, Bronzato Comune It.: 10, Bronzato Comune It. B: 5, Colli Euganei: 10, Ermellinato di Rovigo: 10, Ermellinato di Rovigo B: 10, Nero Italiano: 10, Parma E Piacenza:15 and Romagnolo: 10), 7 Narragansett turkeys and 10 commercial birds. These birds were originally sampled from three different regions (Veneto, Lombardia, and Emilia Romagna) in northern Italy, in many small farms. The original owners of the sampled birds gave their consent to their reuse for research purposes. The final dataset consisted of 180 individuals belonging to 16 populations (Additional file 2, Table S1).

### Mapping and variant calling

Raw sequencing reads were aligned to the turkey reference genome Turkey_5.1 (GCA_000146605.4) using the Burrows-Wheeler Aligner (BWA-mem v.0.7.17) (Li and Durbin, 2009) to generate a BAM file for each individual. All BAM files were sorted and indexed with SAMtools (v.1.9) (Li et al., 2009). Duplicated reads were marked and removed using the Samtools dedup function (Li et al., 2009). Finally, mapping statistics were obtained using Qualimap (Okonechnikov et al., 2016).

Variant Calling was performed using Freebayes software (Garrison and Marth, 2012). Additionally, thresholds set for the variant calling were a minimum base quality of 10, minimum fraction of the alternate allele of 20% and minimum alternate count of 2. The dataset was cleared of samples that had a missing call rate of more than 90%. Bcftools was used for further filtering (Danecek and McCarthy, 2017).

### Data-set preparation and quality control (QC)

For comparison purpose, the genotyping data of (Strillacci et al., 2019; 2020; 2021) were edited using the PLINK 1.9 software (Chang et al., 2015) to remove SNPs with a call rate lower than 99%, SNPs with a minor allele frequency (MAF) lower than 1%, and animals with more than 10% missing genotypes. The variant call format (VCF) files of BAS-APU populations were recoded to plink files using PLINK 1.9 software (Chang et al., 2015). We used the genotype phasing program Beagle (version 5.0) (Browning & Browning, 2007) to loop over each chromosome and confirm allele coding. BCFtools (v.1.9) (Danecek and McCarthy, 2017) was used to concatenate chromosome files to a single VCF file and to index the VCF file of BAS-APU populations. Also, we used BCFtools (v.1.9) (Danecek and McCarthy, 2017) to merge the dataset. A total of 548,744 SNPs passed the quality control.

### Population genetic structure

PLINK 1.9 software (Chang et al., 2015) was used to perform the principal component analysis (PCA) and results were plotted using the ggplot2 R package (Wickham and Wickham, 2016). The ADMIXTURE 1.3 genetic analysis software was used to assess the population genome-wide genetic structure among the populations (Alexander et al., 2009), using different numbers of ancestral populations. The most probable number of ancestral populations was identified in conjunction with the lowest cross-validation error (CV), setting the analysis with an optimal number of clusters (K-value) from 2 to 16, and visualized with BITE V2 R package (Milanesi et al., 2017).

### Identification of ROHs

PLINK 1.9 software (Chang et al., 2015) was used to detect ROH, applying the following parameters to define the ROH: –homozyg-kb 1000 –homozyg-window-missing 2 –homozyg-window-threshold 0.05 –homozyg-window-het 1 –homozyg-window-snp 100 –homozyg-snp 100 –homozyg-density 100 –homozyg-gap 1000. We utilized the R package detectRUNS to visualize the results (Biscarini et al., 2018). ROHs were divided into 5 classes lengths: < 2 Mb, 2 to 4 Mb, 4 to 8 Mb, 8 to 16 Mb, and >16 Mb. For each ROH length category, we calculated the total number of ROH and the average length of an ROH per population. Besides, we calculated the mean sum of ROH per population by summing all ROH values per bird in that category and averaging this per population.

### Genetic diversity indices

PLINK 1.9 software (Chang et al., 2015) was used to estimate the observed and expected heterozygosity (HO, HE), and minor allele frequency (MAF). The median for each population was estimated through the R (R Development Core Team, 2017).

### Inbreeding coefficients

Genomic inbreeding coefficients (*F*_ROH_) were detected using PLINK 1.9 software (Chang et al., 2015). *F*_ROH_ is calculated based on the ratio between the total length of all ROH detected in a sample and the genome length covered by SNPs (in our study on chromosomes 1 to 30, corresponding to 100,100,000 bp). Also, genomic inbreeding coefficients for all birds (F_HOM_) were estimated using the following equation F_HOM_ = (O−E)/(L−E), where O is the number of observed homozygous, E is the number of homozygous expected by chance, and L is the number of genotyped autosomal SNPs, using the PLINK 1.9 software (Chang et al., 2015).

### Detection of selection signatures using iHS

To performed iHS analysis, we divided the studied populations into three groups: the local group includes all Italian local turkey populations, the commercial group includes the commercial line and the Narragansett group includes Narragansett breed. The iHS test was used to identify recent selection signatures within a particular section of the genome. Chromosome-wise haplotype phasing and imputation for missing genotypes were performed using Beagle v5.1 software with default settings (Browning et al., 2018). This test is based on the decay of extended haplotype homozygosity (EHH), computed for ancestral (0) and derived alleles (1) at each core SNP (Voight et al., 2006). The standardized iHS was performed as:

$$iHS=\frac{In\left(\frac{iHH_{A}}{iHH_{D}}\right)-\;E_{p}\;\left[In\left(\frac{iHH_{A}}{iHH_{D}}\right)\right]}{SD_{p}\;\left[In\left(\frac{iHH_{A}}{iHH_{D}}\right)\right]}$$

Where $iHH_{A}$ and $iHH_{D}$ denote the EHH score for ancestral and derived core alleles, respectively. $E_{p}\;\left[In(\frac{iHH_{A}}{iHH_{D}})\right]$ and $SD_{p}\;\left[In(\frac{iHH_{A}}{iHH_{D}})\right]$ represent the expectation and standard deviation within the frequency bin p.

Then, iHS values were estimated for each autosomal SNP and visualized using the rehh package of the R software (Gautier et al., 2017). The iHS score for each SNP was transformed into two-sided p-value as: $\text{piHS}=\;-\;\log_{10}\left[1-2|\Phi (\text{iHS})-0.5|\right]$, where Φ(iHS) is the cumulative Gaussian distribution function of iHS (Gautier et al., 2017). We partitioned the genome into 1 Mb windows with a 500-kb overlap, and the highest |iHS| value within each window was used as the test statistic. The windows that fell within the top 1% of the empirical distribution were identified as selection signature regions.

The full list of turkey genes annotated according to the most recent reference genome was downloaded from Ensemble online Database (Ensemble GTF Release: 111). Genes with an official gene name, ensemble ID associated both with a protein coding and official gene name were catalogued within the detected iHS values using the “intersectBed” command of BEDTools software (Quinlan and Hall, 2010).

## Results

### Population genetic structure

**Fig. 1:**
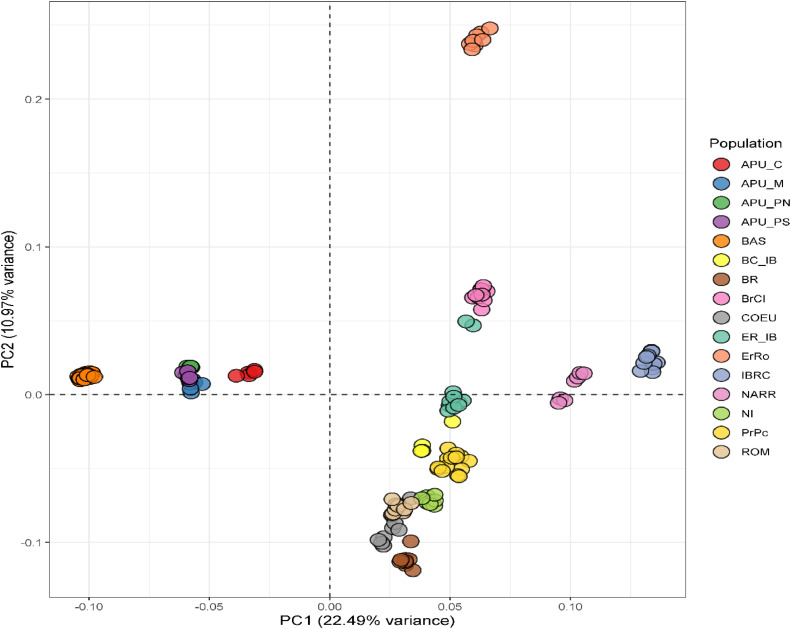
Principal component analysis among 16 Italian turkey populations. Apulian C (APU-C), Apulian M (APU_M), Apulian PN (APU_PN), Apulian PS (APU_PS), Basilicata (BAS), Brianzolo (BR), Bronzato Comune It. (BrCI), Bronzato Comune It. B (BC_IB), Colli Euganei (COEU), Ermellinato di Rovigo (ErRo), Ermellinato di Rovigo B (ER_IB), Commercial Line (IBRC), Narragansett (NARR), Nero Italiano (NI), Parma E Piacenza (PrPc) and Romagnolo (ROM).

The PCA results (Fig. 1) supported close relationships within the southern populations and indicated a clear distinction between the southern and northern populations. PCA1 explains 22.49% of the genetic variance among populations. Results show that the BAS population and APU populations are clearly separate from northern Italian turkey populations, thus forming two clusters. There is an overlap between the APU_M, APU_PN, and APU_PS populations, indicating a close relationship with the BAS population and possible admixture of these populations from the same region in southern Italy. PCA2 explains 10.97% genetic variance, separating the ErRO population from all other populations. IBRC and NARR breeds are closely related, and they are separated from the northern populations. Also, the BrCI population is separated from the other northern Italian populations. The BR, COEU, ER_IB, NI, PrPc, and ROM populations are closely related, and there is partial overlap between the COEU and ROM populations.

**Fig. 2:**
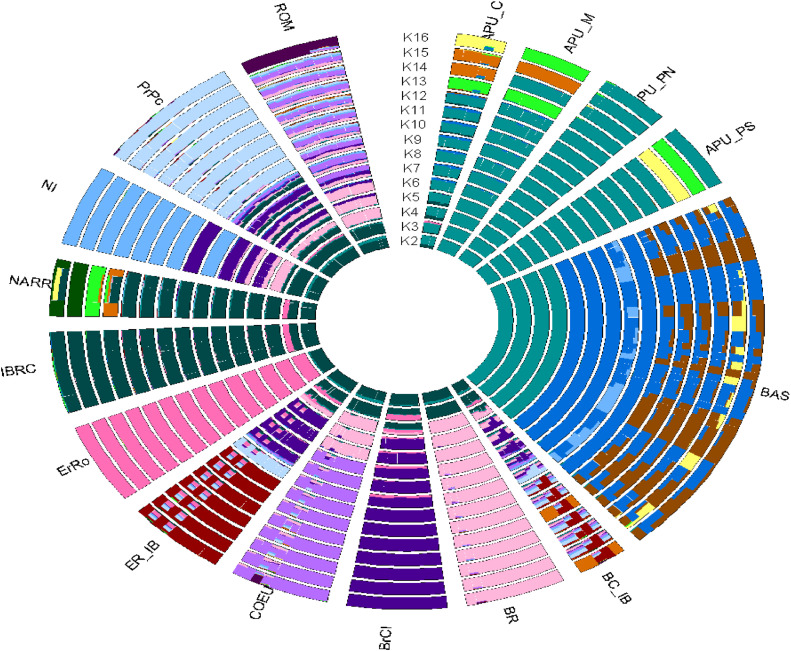
Admixture circle plot from K = 2 to 16 clusters for 16 Italian turkey populations. Apulian C (APU-C), Apulian M (APU_M), Apulian PN (APU_PN), Apulian PS (APU_PS), Basilicata (BAS), Brianzolo (BR), Bronzato Comune It. (BrCI), Bronzato Comune It. B (BC_IB), Colli Euganei (COEU), Ermellinato di Rovigo (ErRo), Ermellinato di Rovigo B (ER_IB), Commercial Line (IBRC), Narragansett (NARR), Nero Italiano (NI), Parma E Piacenza (PrPc) and Romagnolo (ROM).

Admixture analyses were carried out with ancestral components (K) ranging from 2 to 30. The lowest cross-validation (CV) error was calculated to detect the optimal number of ancestral populations. The optimal CV error was identified with K = 16 (Additional file 1, Fig. S1). The Admixture analysis results (Fig. 2) are consistent with the PCA. The inferred population structure for K = 2 separated the BAS-APU populations from the northern populations. At K = 6, the BAS population separated from APU_C, APU_M, APU_PN, and APU_PS populations, and there is overlap among APU_M, APU_PN, and APU_PS populations, confirming the findings of the PCA. At K = 3, the ErRo population separated from the northern populations. There was no variation between IBRC and NARR populations from K = 2 to 12, which indicates a close genetic relationship, supporting the PCA results. The fourth ancestral component separated the BR population from the northern populations, and the fifth ancestral component separated the BrCI population from other northern populations. In general, closer genetic relationships were observed between COEU and ROM populations, confirming the PCA analyses.

### Runs of homozygosity

A total of 14,390 ROHs were identified in the 16 turkey populations, and the average number of ROH segments per individual ranged from 42.8 to 131. These ROH segments mean length ranged from 1.8 to 13.3 Mb for the studied populations. The highest number of ROHs was observed in the BAS population, while the lowest was observed in the APU_C population (Table 2; Additional file 2, Tables S2, S3, and S4). The BAS population showed the highest number of ROHs (> 4 Mb), in contrast to the IBRC populations. The ROH analysis confirmed that ROH segments ranging from 0 to 2 Mb were prevalent across all populations. Furthermore, our findings revealed that ROH segments ranging from 2 to 4 Mb are comparatively prevalent in the Italian populations genomic landscape, particularly in the BAS and COEU populations. These results identify differences among populations in the distribution of ROHs, providing insight into the genomic diversity of these populations.

**Table 2:** Summary of the number of runs of homozygosity (Mb) per class size in each population.

Pop*/ Class (Mb)	0-2	2-4	4-8	8-16	>16	Total
APU_C	100	26	19	8	61	214
APU_M	133	107	61	32	74	407
APU_PN	145	77	59	32	70	383
APU_PS	145	89	52	29	85	400
BAS	1003	753	394	224	403	2777
BC_IB	301	213	109	27	5	655
BR	527	345	163	61	16	1112
BrCI	474	318	198	110	28	1128
COEU	631	372	203	70	12	1288
ER_IB	449	238	110	27	9	833
ErRo	330	288	246	164	102	1130
IBRC	407	130	28	1	NA	566
NARR	462	283	100	25	2	872
NI	462	209	118	32	6	827
PrPc	598	243	98	37	4	980
ROM	538	211	56	12	1	818
Total	6705	3902	2014	891	878	14390

*Pop, population; Apulian C (APU-C), Apulian M (APU_M), Apulian PN (APU_PN), Apulian PS (APU_PS), Basilicata (BAS), Brianzolo (BR), Bronzato Comune It. (BrCI), Bronzato Comune It. B (BC_IB), Colli Euganei (COEU), Ermellinato di Rovigo (ErRo), Ermellinato di Rovigo B (ER_IB), Commercial Line (IBRC), Narragansett (NARR), Nero Italiano (NI), Parma E Piacenza (PrPc) and Romagnolo (ROM).

### Genetic diversity indices

The results of genetic diversity indices are presented in Table 3. The highest averages for Ho (0.173), He (0.128), and MAF (0.099) were observed in APU_C, while the lowest means for Ho (0.01) were seen in the APU_M population, and He (0.071) and MAF (0.052) in the BAS population.

**Table 3:** Genetic diversity indices and inbreeding coefficient for the Italian local turkey populations.

Population	Ho±SD	He±SD	MAF±SD	*F*_ROH_ ±SD
APU_C	0.173±0.287	0.128±0.198	0.099±0.159	0.462±0.010
APU_M	0.01±0.213	0.085±0.168	0.066±0.138	0.593±0.027
APU_PN	0.105±0.224	0.085±0.169	0.065±0.136	0.592±0.041
APU_PS	0.116±0.239	0.085±0.169	0.065±0.134	0.561±0.040
BAS	0.075±0.162	0.071±0.15	0.052±0.119	0.604±0.076
BC_IB	0.171±0.213	0.217±0.191	0.158±0.157	0.365±0.125
BR	0.183±0.249	0.153±0.198	0.117±0.164	0.326±0.108
BrCI	0.177±0.206	0.19±0.199	0.143±0.166	0.408±0.042
COEU	0.163±0.187	0.164±0.172	0.114±0.137	0.364±0.184
ER_IB	0.25±0.234	0.234±0.189	0.172±0.161	0.215±0.121
ErRo	0.076±0.166	0.078±0.16	0.059±0.13	0.643±0.028
IBRC	0.391±0.223	0.337±0.154	0.256±0.148	0.096±0.018
NARR	0.235±0.23	0.261±0.199	0.2±0.172	0.294±0.137
NI	0.23±0.283	0.185±0.213	0.147±0.181	0.211±0.118
PrPc	0.275±0.229	0.259±0.197	0.198±0.171	0.157±0.074
ROM	0.251±0.245	0.22±0.198	0.165±0.167	0.160±0.027

Ho, observed heterozygosity; He, expected heterozygosity; MAF, average minor allele frequency; FROH, genomic inbreeding coefficient based on run of homozygosity. Apulian C (APU-C), Apulian M (APU_M), Apulian PN (APU_PN), Apulian PS (APU_PS), Basilicata (BAS), Brianzolo (BR), Bronzato Comune It. (BrCI), Bronzato Comune It. B (BC_IB), Colli Euganei (COEU), Ermellinato di Rovigo (ErRo), Ermellinato di Rovigo B (ER_IB), Commercial Line (IBRC), Narragansett (NARR), Nero Italiano (NI), Parma E Piacenza (PrPc) and Romagnolo (ROM).

### Genomic inbreeding coefficient

**Fig. 3:**
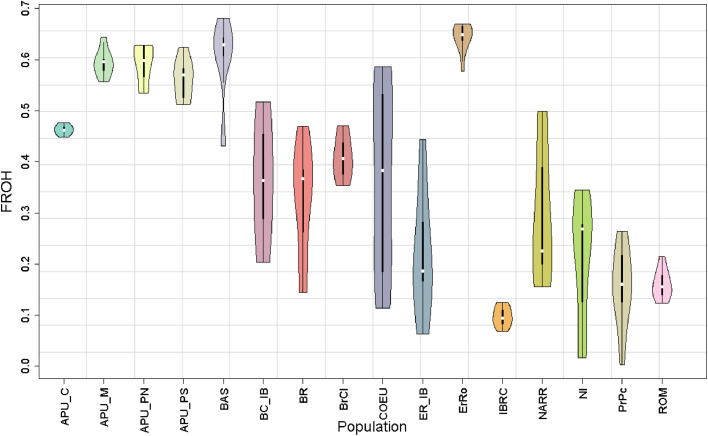
Box plot of the inbreeding coefficient based on ROH segments in all populations. Apulian C (APU-C), Apulian M (APU_M), Apulian PN (APU_PN), Apulian PS (APU_PS), Basilicata (BAS), Brianzolo (BR), Bronzato Comune It. (BrCI), Bronzato Comune It. B (BC_IB), Colli Euganei (COEU), Ermellinato di Rovigo (ErRo), Ermellinato di Rovigo B (ER_IB), Commercial Line (IBRC), Narragansett (NARR), Nero Italiano (NI), Parma E Piacenza (PrPc) and Romagnolo (ROM).

Genomic inbreeding coefficients (F_ROH_) were evaluated based on ROH for each separate population (Table 3; Fig. 3). The F_ROH_ values for each population ranged from 0.096 to 0.643. The F_ROH_ values were 0.462, 0.593, 0.592, 561 and 0.602 in APU_C, APU_M, APU_PN, APU_PS and BAS populations, respectively. The ErRo population had the highest inbreeding coefficient value (F_ROH_ = 0.643), followed by the BAS population (F_ROH_ = 0.604), while the lowest inbreeding coefficient value was observed in the IBRC breed (F_ROH_ = 0.096). The inbreeding coefficients based on the difference between the observed and expected numbers of homozygous genotypes (F_HOM_) were estimated for all studied populations. The estimates of F_HOM_ were 0.389, 0.648, 0.631, 591 and 0.731 in APU_C, APU_M, APU_PN, APU_PS and BAS populations, respectively (Additional file 1; Fig. S2). The highest F_HOM_ value (0.73) was noticed in the ErRo populations, while the lowest value (-0.36) was observed in the IBRC breed.

### Signatures of selection based on iHS

**Fig. 4:**
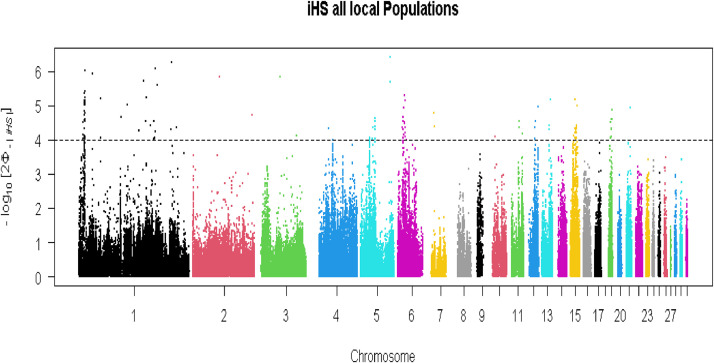
Manhattan plot of the genome-wide iHS analysis within local group.

**Fig. 5:**
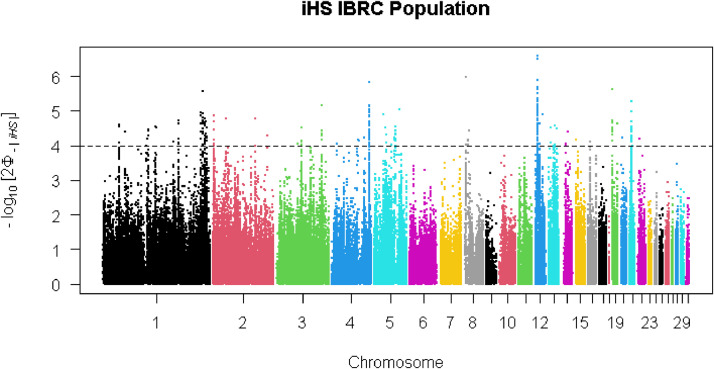
Manhattan plot of the genome-wide iHS analysis within commercial line.

**Fig. 6:**
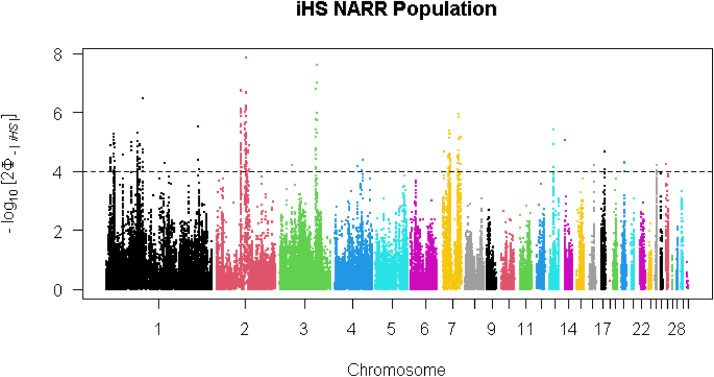
Manhattan plot of the genome-wide iHS analysis within Narragansett breed.

The top 1% of iHS revealed 102 SNPs representing putative selection signatures [−log10(p-value) > 4] within the local group (Fig. 4). These SNPs were defined in 20 candidate genomic regions across eight chromosomes that putatively have been under positive selection. A total of 73 genes were detected within these genomic regions including, *FAM107B, FRMD4A, MSTN, SOX30, MIGA2* and *CRAT* (Additional file 2, Table S5). In the commercial line, 167 SNPs and 48 genes were distributed in 19 genomic regions across eight chromosomes that showed strong evidence of selection (Fig. 5; Additional file 2, Table S6). These genes including *PDZRN4, MAL2, CCN3* and *MMP13*, were identified in eight genomic regions on chromosomes 1 and 3. A total of 264 SNPs and 90 genes were identified in 27 genomic regions in eight chromosomes as selection signals in the NARR breed (Fig. 6; Additional file 2, Table S7). The candidate genes *HSF2, GJA1* and *FKBP7* were in selected regions on chromosomes 2 and 7 of the Narragansett breed, and these genes were considered as important candidate genes for heat stress. The identified candidate genes using iHS method in the three groups (local group, commercial line and Narragansett breed) were related to for economic traits e.g. adaptation to heat stress.

## Discussion

### Population genetic structure

We performed a PCA and Admixture analysis to comprehend the population structure and genetic relationships. Remarkably, the southern turkeys were separated from the northern populations according to the geographic distribution, as presented in PCA (Fig. 1). The results showed that the genetic background of the BAS and APU populations is different from the northern turkey populations. For the northern population, the IBRC, ErRo, and BrCI populations are genetically distant from the rest of the northern populations. Bernini et al. (2021) found that the ErRo and BrCI populations were separated from BR, ROM, PrPc, NI, and COEU populations. The closeness of the relationship between BAS and APU populations could be related to the geographical origin of these populations in the southern regions of Italy. These results are also supported by the ADMIXTURE findings, which indicate different composition backgrounds for the northern and southern Italian turkeys (Fig. 2). Bernini et al. (2021) stated that the proportion of ancestors found in the ROM population using the ADMIXTURE analysis was inversely proportional to the geographical distance from Veneto, Emilia Romagna, and Lombardia.

### Runs of homozygosity

The ROH analysis emphasizes crucial novel marker-based information in halting future diversity loss. The increasing homozygosity in certain genomic segments of poultry is a result of several factors, including population bottlenecks, genetic drift, and inbreeding, leading to a high frequency of ROH and affecting the genetic diversity within these populations and breeds. Several studies have detected ROH in turkeys and their relationship with inbreeding coefficients, especially correlated to the economic traits in different turkey breeds (Marras et al. 2018; Bernini et al. 2021). Nevertheless, the distribution and patterns of ROH in the native Italian turkey populations are still completely unknown. The distribution of the number and length of ROH can reflect the genetic diversity within the studied populations. The BAS population had the highest number and length of ROH among all studied populations, while the COEU, ErRo, BR, and BrCI turkey populations had higher numbers and lengths of ROH than the APU populations, ROM, BC_IB, ER_IB, IBRC, NARR, NI, and PrPc (Table 2). The detection of ROH could be helpful for conservation strategies since birds with high levels of F_ROH_, as noticed in the BAS population, can be excluded or given less emphasis for mating purposes in endangered populations to limit the loss in genetic diversity and promote genetic diversity. The differences in the number and length of ROHs could be ascribed to factors like geographic location, or genetic makeup for certain characteristics specific to each population and indicating adaptation to environmental conditions. Longer geographic isolation and the result of early historical inbreeding that produced regions (McHugo et al. 2019), which collapsed due to recombination. Geographic location, along with small-scale farming practices, likely subjected the studied turkey populations to various environmental stresses. These pressures may have influenced their fitness, leading to adaptive changes over time through shifts in the frequency of beneficial or detrimental alleles (Mastrangelo et al., 2023). The BAS population revealed a higher number of long ROH, suggesting high recent inbreeding. Long ROH are common in local breeds of chicken, which is consistent with the limitations on efficient genetic management brought about by the lack of breed registration and pedigree information (Bortoluzzi et al. 2018; Cendron et al. 2021; Zhang et al. 2023). Marras et al. (2018) reported an average of 126.21 ROH per turkey and an average ROH length of ∼1.7 Mb in commercial turkey breeds. Strillacci et al. (2020) found that the percentage of ROH within length classes is similar in Mexican turkeys and commercial breeds. However, the number of ROH and the mean ROH length per sample varied between Mexican populations. The commercial breeds also appeared to be less variable in terms of ROH compared to Mexican turkeys. Adams et al. (2021) found that the average number of ROH per individual ranged from 81.68 to 87.14 for all lines, with values ranging from 20 to 118 for line A, 2 to 110 for line B, and 40 to 108 for line C. Both commercial and local breeds may have considerable levels of inbreeding despite having different management types. The findings of this study agree with the results of Bernini et al. (2021), who studied ROH in seven Italian local turkey populations. Also, Bernini et al. (2021) stated that the ErRo is the only population that showed a clear homozygosity state and ROH islands for all birds.

### Genetic diversity indices

In the current study, we used genomic SNP data to reveal genetic diversity in Italian turkey populations. Enhancing our understanding of the population structure and within-breed diversity in animal species is essential for maximizing breed selection, comprehending environmental adaptation, and carrying out conservation strategies (Bernini et al. 2021; Cendron et al. 2021). Heritage turkey populations now constitute a distinct genetic biodiversity pool. Since indigenous populations have unique characteristics, such as the ability to adapt to extended cultivation in harsh environments, the in-situ conservation of these populations is absolutely vital. The genetic characterization and population structure of these populations are important steps in proving the efficacy of a preservation strategy (Baes et al. 2019; Bernini et al. 2021). Genetic diversity indices, which are key parameters in the genetic management of populations, were used to detect the levels of genetic variation within the populations. Since the Ho, He, and MAF estimates are low in the APU M, BAS, ErRo, APU PN, and APU_PS populations, the lowest genetic diversity was noticed for these populations. In contrast the highest estimates were observed in the IBRC line. This may be a result of population bottlenecks caused by keeping these populations in a rather small demographic space. The lower heterozygosity values observed in the APU M, BAS, ErRo, APU PN, and APU PS populations, compared to other populations, highlight the challenges in maintaining biodiversity within these populations. Consequently, it is crucial to focus on the preservation of these populations. Implementing targeted genetic conservation programs, such as breeding schemes aimed at increasing genetic diversity, controlling inbreeding, and promoting both in-situ and ex-situ conservation strategies, would be beneficial for these breeds (Cendron et al., 2021). The Ho, He, and MAF values agree with the findings mentioned in the previous study on Italian turkeys (Bernini et al. 2021). Conversely, the average Ho and He values were different from those found in a prior study of Canales et al. (2023) using microsatellite markers, where the authors demonstrated higher values for 10 domestic turkey breeds from different countries.

### Genomic inbreeding coefficient

Genome-wide SNPs are especially helpful for identifying genomic regions with decreased heterozygosity (Bortoluzzi et al. 2018). The ability to compute genomic inbreeding coefficients is helpful when pedigree information is unavailable, which is often the case in poultry species. This is crucial for local and threatened populations, such as the indigenous Italian turkey populations. We observed significant differences within populations for inbreeding with F_ROH_ estimates ranging from 0.096 to 0.643 for the Italian turkey populations (Fig. 3). The highest F_ROH_ and F_HOM_ values were observed in the APU M, BAS, ErRo, APU PN, and APU_PS populations, while the lowest value was observed in IBRC line. The high level of inbreeding in these populations is likely due to closed breeding in small sizes and imperfect management. These findings demonstrate how breeding strategies and selection had an uneven impact on the genetic composition of the studied populations, for example the IBRC line. These results underscore the urgent need for targeted efforts to preserve turkey genetic diversity and reverse the decline of their populations. Implementing genetic conservation and management strategies is essential to mitigate the negative effects of inbreeding and promote the sustainable development of these turkey populations. The same trends were observed in previous studies conducted by Strillacci et al. (2020) and Bernini et al. (2021). Marras et al. (2018) found that the F_ROH_ values ranged from 0.014 to 0.44 in commercial turkey populations, and Adams et al. (2021) reported that the F_ROH_ values ranged from 0.26 to 0.28 in three purebred turkey lines.

### Signatures of selection based on iHS

To identify selection signals, we applied the iHS method to the top 1% of high-frequency SNPs within the three groups. Based on haplotype data, iHS is a main technique for identifying positive selection within populations. To find potential selective signals, this research concentrated on these top SNPs. In the local group that includes all local populations, we identified several genes within four genomic regions that might have been under selection: *FAM107B* and *FRMD4A* genes on chromosome 1 (7.11-7.46Mb), *MSTN* on chromosome 7 (7 -7.88Mb), *SOX30* on chromosome 15 (10.51-11.22Mb), and *MIGA2* and *CRAT* on chromosome 19 (5.26-6.18Mb). The *FAM107B* (family with sequence similarity 107 member B) gene has response to changes in heat stress levels and plays a role in heat-shock induction (Yonekura et al., 2010). Genome-wide association studies have shown that the *FAM107B* gene is significantly associated with heat stress response in sheep and dairy cattle (Saadatabadi et al., 2021; Luo et al., 2021). Another important gene was found in a region under potential selection. Myostatin (*MSTN*) is a key regulator of skeletal muscle growth (Beyer et al., 2013). The *MSTN* gene is highly conserved among mammalian species, and acts in a nearly unique way to reduce muscle size. *MSTN*-deficient animals display an increase in skeletal muscle mass known as double muscling. Ye et al. (2007) and Zhang et al. (2019) found that SNPs of *MSTN* gene were associated significantly with growth, carcass traits, mortality, blood oxygen and hen antibody titer to infectious bursal disease virus vaccine in broiler lines. Zhang et al. (2012) reported that the AA and GA genotypes were higher than GG genotype for body weight from 6 to 18 weeks of age in Bian chicken. The overexpression of *MSTN* was observed at 2 weeks of age in broilers and laying hens (Bhattacharya et al., 2015). Interestingly, we detected three genes under positive selection in the commercial line, *PDZRN4* on chromosome 1 (28.401-28.404Mb), and *MAL2* and *CCN3* on chromosome 7 (80.64-80.69Mb). The *PDZRN4* gene is known to play roles in protein degradation pathways (Czech et al., 2023). The *PDZRN4* gene has a potential importance in heat stress tolerance in cattle and provides valuable genetic markers for selection programs aimed at improving heat stress tolerance (Czech et al., 2023; Shi et al., 2024). Furthermore, *PDDRN4* was found to be an important gene correlated with poor sperm motility in Holstein-Friesian bulls (Hering et al., 2014). Wang et al. (2021) found that this gene can affect fat metabolism in pigs. Candidate genes *HSF2* and *GJA1* on chromosome 2 (60.46-60.93), and *FKBP7* on chromosome 7 (14.03-15Mb) were in regions containing selective sweeps revealed by iHS approach in the Narragansett breed. The *HSF2* gene was detected by iHS analysis in a region under selection signature in the Narragansett breed. Heat shock proteins (HSPs) are regulated by a family of transcription factors known as heat shock factors (HSFs), with *HSF2* being the most extensively studied member in mammals (Joutsen and Sistonen, 2019). Notably, *HSF2* is essential for cell survival during chronic accumulation of misfolded proteins, highlighting its distinct role in cellular stress responses (Joutsen et al., 2020). Beyond stress responses, *HSF2* also plays crucial roles in various physiological processes, including embryogenesis, corticogenesis, and spermatogenesis (Abane and Mezger, 2010; Joutsen and Sistonen, 2019). The *GJA1* gene, identified in a region under positive selection, is a functional gene associated with chicken breast muscle growth. This discovery may reveal a new functional role for *GJA1* in pectoral muscle development, expanding its known function in skeletal form (Watkins et al., 2011). Additionally, genome-wide association studies have linked *GJA1* to chest muscle weight and chest muscle proportion (Liu et al., 2013). Aboelhassan et al. (2023) reported that the expression of *GJA1* gene was higher in Mamourah breed and was correlated with increased breast weight. Furthermore, the overexpression of *GJA1* gene in Mamourah, Inshas and Leghorn breeds was found to be associated with higher thigh weight. Scientific research on selective sweeps in turkey populations is still limited. To our knowledge, this represents the first genome-wide scan of potential selection signatures using the iHS method in Italian local turkey group. Remarkably, these genes play a crucial role for heat stress tolerance, growth, and carcass traits in Italian local populations, which could serve as a foundation for conservation and breeding programs especially for heat stress in these populations. In the commercial line there is also evidence of selective sweeps for thermo-tolerance and fat metabolism. The identified candidate genes are considered as a strong selection signature related to body weight form and tolerance to heat stress in Narragansett breed. Aslam et al. (2014) discovered selection signatures in 48 genomic regions in commercial turkey breeds, and 6 significant regions in local turkey breeds. These regions ranged from 1.5 Mb to 13.8 Mb and are located on 14 different chromosomes. Bernini et al. (2021) detected a selection signature based on ROH in a genomic homozygous region on chromosome 10 for characteristics associated with survival, such as reproduction efficiency across Italian turkey populations. Durosaro et al. (2022) found that purifying selection was the dominant selective force across all color types for native Nigerian turkeys and United British turkeys. This purifying selection indicates selection against newly developing harmful mutations in the feather color gene, as well as for the maintenance of feather color's biological activities such as protection, adaptation, and signaling (Brunet et al. 2021). Mastrangelo et al. (2023) detected selective sweeps in 8 genomic regions across seven chromosomes and distributed: four regions for the local chicken breeds ranging from 460 kb on chromosome 8 to 550 kb on chromosome 25, two regions for the heavy breeds, and one region for the northern and one region for the southern Italian populations. Also, they reported that the candidate genes within the selective sweeps on *GGA1* were associated with cold adaptability and heat stress in northern and southern Italian chicken breeds.

## Conclusions

This study reveals insights into the genomic diversity and structure of Italian local turkey populations. Our findings are mostly consistent with the breeding history of the studied populations. The populations have maintained unique characteristics, likely due to differences in genetic origin, environment, genetic isolation, and inbreeding. The BAS, APU_M, APU_PN, and APU_PS populations showed lower genetic variability compared to the other studied populations. The identified genomic regions and selective sweeps suggest that selective breeding and genetic drift had a strong influence on the frequency of genes controlling growth traits, such as carcass and meat quality, reproductive traits, and metabolic traits. These findings are important for developing and implementing future conservation plans. For example, genetic diversity analysis can facilitate the creation of focused mating strategies that safeguard the most vulnerable populations.

## Declaration of competing interest

We, the undersigned authors of the manuscript declare that we have no conflicts of interest related to the research, authorship, and/or publication of this article.

## Appendix Supplementary materials

Image, application 1

Image, application 2
