# Supplementary material for: Genome-wide analyses of genomic diversity, population structure and selection signatures in Italian turkey populations
Source: Poult Sci. 2024 Nov 20;104(1):104543. doi: 10.1016/j.psj.2024.104543 (PMC11647235; doi:10.1016/j.psj.2024.104543)
Supplement: Supplementary file 1 [file mmc1.docx]

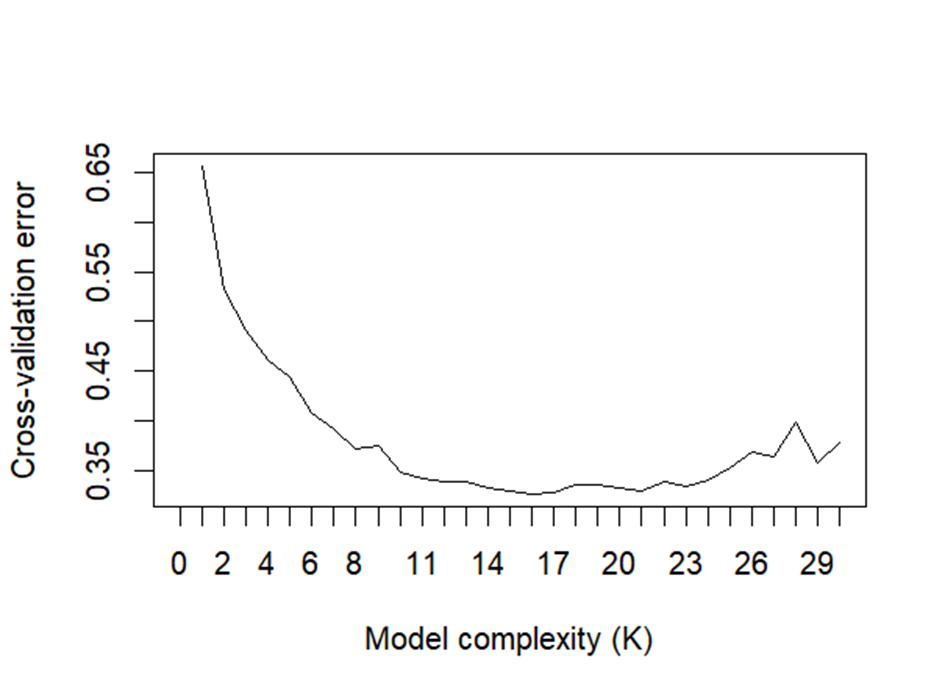


Figure S1. Cross validation error


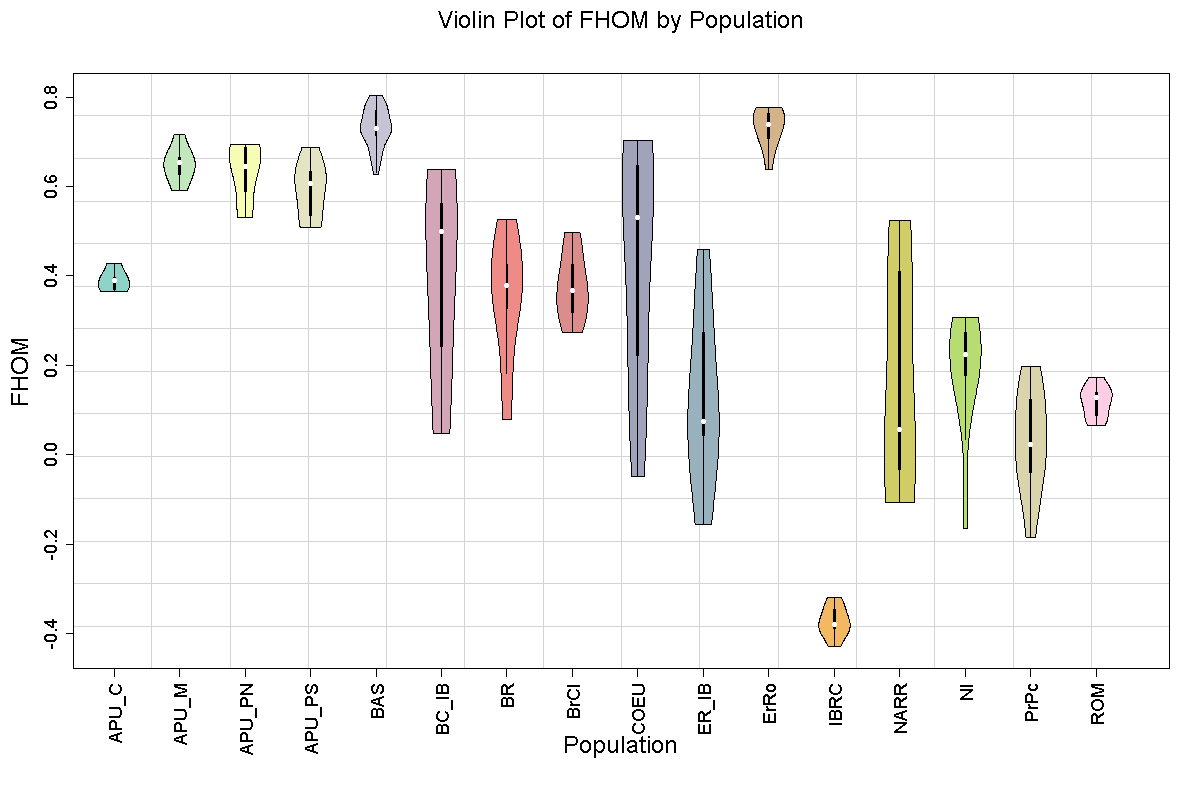


Figure S2. Inbreeding coefficients based on the difference between the observed and expected numbers of homozygous genotypes. Apulian C (APU-C), Apulian M (APU_M), Apulian PN (APU_PN), Apulian PS (APU_PS), Basilicata (BAS), Brianzolo (BR), Bronzato Comune It. (BrCI), Bronzato Comune It. B (BC_IB), Colli Euganei (COEU), Ermellinato di Rovigo (ErRo), Ermellinato di Rovigo B (ER_IB), Commercial Line (IBRC), Narragansett (NARR), Nero Italiano (NI), Parma E Piacenza (PrPc) and Romagnolo (ROM).
